# The triglyceride-glucose index predicts ischemic heart disease risk in Koreans: a prospective study using National Health Insurance Service data

**DOI:** 10.1186/s12933-020-01186-2

**Published:** 2020-12-10

**Authors:** Byoungjin Park, Yong-Jae Lee, Hye Sun Lee, Dong-Hyuk Jung

**Affiliations:** 1 Department of Family Medicine, Yonsei University College of Medicine, Yongin Severance Hospital, 363 Dongbaekjukjeondae-ro, Giheung-gu, Yongin-si, Gyeonggi-do 16995 Republic of Korea; 2 Department of Family Medicine, Yonsei University College of Medicine, Seoul, 03722 Republic of Korea; 3 Department of Family Medicine, Gangnam Severance Hospital, Seoul, 06273 Republic of Korea; 4 Biostatistics Collaboration Unit, Department of Research Affairs, Yonsei University College of Medicine, Seoul, 06273 Republic of Korea

**Keywords:** Triglyceride glucose index, Early insulin resistance, Prospective cohort study, Incident ischemic heart disease

## Abstract

**Background:**

Ischemic heart disease (IHD) without diabetes is considered an important challenge to human health and is associated with a poor prognosis, as well as a lack of health awareness. We prospectively investigated the relationship between the triglyceride-glucose (TyG) index, a surrogate marker of early insulin resistance, and incident IHD risk in a large cohort of nondiabetic Korean adults using National Health Insurance Service data.

**Methods:**

We assessed 16,455 participants (8426 men and 8029 women) without diabetes using data from a health risk assessment study (HERAS) and Korea Health Insurance Review and Assessment (HIRA) data. The participants were divided into four groups according to TyG index quartiles, calculated as ln [fasting triglycerides (mg/dL) × fasting plasma glucose (mg/dL)/2]. We prospectively assessed hazard ratios (HRs) with 95% confidence intervals (CIs) for IHD using multivariate Cox proportional-hazards regression models over a 50-month period that followed the baseline survey.

**Results:**

During the follow-up period, 322 (2.0%) participants developed IHD. HRs of IHD for TyG index quartiles 2–4 were 1.61 (95% CI 1.05–2.48), 1.85 (95% CI 1.21–2.81), and 2.29 (95% CI 1.50–3.51), respectively, after adjusting for age, sex, body mass index, smoking status, alcohol intake, and physical activity.

**Conclusions:**

A higher TyG index precedes and significantly predicts future IHD among nondiabetic Koreans. Accordingly, the TyG index may be a useful measure in assessing cardiovascular risk for nondiabetic adults in the preclinical stage.

## Background

Ischemic heart disease (IHD) is a source of premature morbidity and mortality among middle-aged and older individuals, and it is an important challenge to human health in both developing and developed countries [1]. The burden of premature IHD in an ageing population cannot be underestimated, as it is a factor that decreases quality of life and increases social burden [2]. Accordingly, assessing and identifying potential risks for IHD in the preclinical stage is worthwhile, facilitating disease prevention and slowing the progression of IHD.

Accumulating evidence suggests that the triglyceride-glucose (TyG) index, a simple and widely accessible measure, is a novel and surrogate marker for early insulin resistance [3, 4]. Epidemiological studies conducted in the Korean population have shown that the TyG index is a better indicator of metabolic syndrome and type 2 diabetes than the homeostasis model assessment of insulin resistance (HOMA-IR) [5, 6]. Koreans comprise a group of East Asians of ethnic homogeneity, with lower overall body mass index (BMI) values and much higher proportions of carbohydrate intake than Westerners [7]. The prevalence of both hypertriglyceridemia (≥ 150 mg/dL) and impaired fasting glucose is reportedly 20–30% among Korean adults, contributing to increased risks of coronary heart disease, according to data from the Korea National Health and Nutrition Examination Survey (KNHANES) [8, 9].

Prospective studies of predictive values of the TyG index for cardiovascular diseases (CVD) have primarily focused on pre-existing coronary arterial disease or diabetes mortality [10, 11], and the TyG index has been shown to be associated with subclinical atherosclerosis symptoms, such as arterial stiffness and preclinical coronary arterial calcification [12–14]. Accordingly, since nondiabetic individuals with IHD tend to exhibit poorer prognosis than diabetic patients without IHD [15, 16], we prospectively investigated potential relationships between the TyG index and IHD incidence within a large-scale, community-dwelling, nondiabetic adult cohort using National Health Insurance Service data.

## Methods

### Study participants

**Fig. 1:**
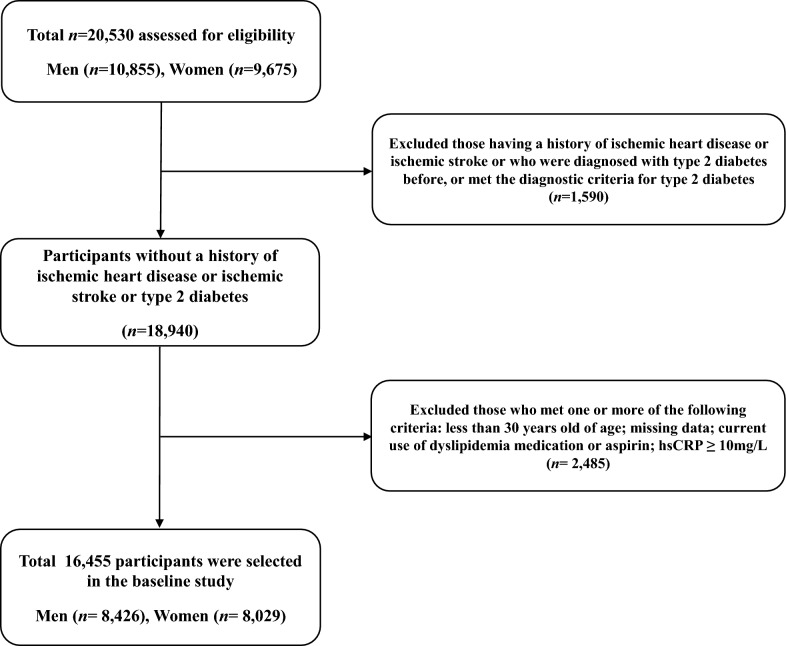
Flowchart for the selection of study participants

This study is based on a health risk assessment study (HERAS) that aimed to characterize cardiovascular risk factors and to explore surrogate markers of CVD in Korean adults. The study cohort consisted of 20,530 individuals aged ≥ 20 years who voluntarily visited the Health Promotion Center of Gangnam Severance Hospital, Yonsei University College of Medicine for regular health examinations between November 2006 and June 2010. Among 20,530 participants initially assessed, 1,590 (7.7%) participants with a history of IHD or ischemic stroke, a previous diagnosis of type 2 diabetes, or a fasting plasma glucose level ≥ 126 mg/dL [17] were excluded. We also excluded participants who met at least one of the following criteria: age < 30 years, missing data, current use of dyslipidaemia medication or aspirin, or high-sensitivity C-reactive protein (hsCRP) levels ≥ 10 mg/L (*N* = 2485). After exclusion criteria were applied, 16,455 participants (8426 men and 8029 women) were included in our final analysis (Fig. 1).

### Data collection

Each participant completed a lifestyle and medical history questionnaire that included information regarding cigarette smoking, alcohol consumption, and physical activity. Smoking status was defined using the following categories: non-smoker, ex-smoker, and current smoker. Questions regarding alcohol intake included information regarding consumption frequency on a weekly basis. Regular alcohol consumption was defined as alcohol consumption ≥ 140 g per week. Participants were asked about their levels of physical exercise on a weekly basis, and regular exercise was defined as physical activity of moderate intensity ≥ three times per week. Body weight and height were measured to the nearest 0.1 kg and 0.1 cm, respectively, in light indoor clothing without shoes. BMI was calculated as an individual’s weight in kilograms divided by the square of his/her height in metres (kg/m^2^). Systolic blood pressure and diastolic blood pressure were measured on the patient’s right arm using a standard mercury sphygmomanometer in the sitting position after 10 min of rest (Baumanometer, W.A. Baum Co Inc., Copiague, NY, USA). All blood samples were obtained from the antecubital vein after overnight fasting for 12 h. Fasting plasma glucose, total cholesterol, triglyceride, and high-density lipoprotein (HDL) cholesterol levels were measured via enzymatic methods using a Hitachi 7600 automated chemistry analyser (Hitachi Co.; Tokyo, Japan). hsCRP concentrations were measured with a Roche/Hitachi 912 System (Roche Diagnostics, Indianapolis, IN, USA) using a latex-enhanced immunoturbidimetric method with a low limit of detection of 0.09 mg/L. Hypertension was defined as a systolic blood pressure ≥ 140 mmHg, a diastolic blood pressure ≥ 90 mmHg, or current use of hypertension medication [18]. Chronic kidney disease (CKD) was defined either as renal tissue damage or reduced renal functioning, as determined by an eGFR value < 60 mL/min/1.73 m^2^ or proteinuria 1+ or greater [19].

### Study outcomes

The primary outcome assessed was IHD, which consisted of angina pectoris (ICD-10 code I20) or acute myocardial infarction (ICD-10 code I21) that occurred after initial study enrolment. To define baseline and post-survey outcomes, we linked a personal, 13-digit identification number that was assigned to each subject with Korea Health Insurance Review and Assessment (HIRA) data, which is a repository of claims data collected in the process of reimbursing healthcare providers, between November 2006 and December 2010. Participants that were found to have had IHD or ischemic stroke (ICD-10 codes I20, I21, and I63) at the time of their initial assessment were excluded before the final analysis.

### Statistical analysis

TyG index values were categorised into quartiles as follows: Q1 (≤ 8.08), Q2 (8.09–8.45), Q3 (8.46–8.85), and Q4 (≥ 8.86). All data are presented as means with standard deviations or percentages. The baseline characteristics of the study population according to the TyG index quartiles were compared using an analysis of variance (ANOVA) model for continuous variables and the Pearson’s Chi-squared test for categorical variables. Kaplan–Meier curves were used to assess the cumulative incidence of IHD. The log-rank test was used to determine whether the distributions of cumulative IHD incidence differed among groups. Pairwise comparisons of receiver-operating characteristic (ROC) curves were used to contrast areas under ROC curves (AUC) for IHD incidence based on TyG index, fasting plasma glucose, and serum triglyceride levels. Further, AUC values were used to test the sensitivity and specificity of biomarkers for predicting IHD. In multivariate analysis, after setting the lowest TyG index value quartile as a reference group, hazard ratios (HRs) and 95% confidence intervals (CIs) for incident IHD were calculated using the Cox proportional hazards regression model after adjusting for potential confounding variables [4]. An ex-post power calculation was also performed: for a HR of 1.5, the calculated power was 0.983; for a HR of 2.0, the calculated power was > 0.999. All analyses were performed using SAS version 9.4 software (SAS Institute Inc., Cary, NC, USA). All statistical tests were two-sided, and statistical significance was set at *P* < 0.05.

## Results

Table 1 shows the baseline characteristics of the study population (n = 16,455; 8,426 men and 8,029 women) according to TyG index quartiles. The mean age and BMI of the study population were 46.1 ± 9.5 years and 23.4 ± 3.0 kg/m^2^, respectively. The mean fasting plasma glucose concentration was 91.4 ± 9.8 mg/dL, the mean triglycerides level was 124.2 ± 84.9 mg/dL, and the mean TyG index value was 8.49 ± 0.56. The prevalences of hypertriglyceridemia and impaired fasting glucose were 18.0% and 25.0%, respectively. Older adults, aged 65 years and older, comprised 4.5% of the study population.

**Table 1:** Baseline characteristics of the study population according to TyG index quartiles

Variables	Total n = 16,456	Quartile of TyG index				*P* value^*^	Post hoc^†^
		Q1 n = 4140	Q2 n = 4119	Q3 n = 4096	Q4 n = 4100		
TyG index	8.49 ± 0.56	≤ 8.08	8.09–8.45	8.46–8.85	≥ 8.86	-	-
Age (years)	46.1 ± 9.5	44.0 ± 8.9	46.0 ± 9.5	47.5 ± 9.7	46.8 ± 9.4	< 0.001	a,b,c,d,e,f
Male sex (%)	51.2	27.7	43.3	58.4	75.8	< 0.001	-
Body mass index (kg/m^2^)	23.4 ± 3.0	21.6 ± 2.5	22.7 ± 2.7	23.9 ± 2.8	25.2 ± 2.8	< 0.001	a,b,c,d,e,f
Systolic BP (mmHg)	121.9 ± 15.5	115.0 ± 13.9	119.8 ± 14.8	124.1 ± 14.9	128.8 ± 14.8	< 0.001	a,b,c,d,e,f
Diastolic BP (mmHg)	76.2 ± 10.1	71.5 ± 9.1	74.7 ± 9.5	77.7 ± 9.6	80.9 ± 9.7	< 0.001	a,b,c,d,e,f
Mean BP (mmHg)	91.5 ± 11.5	86.0 ± 10.3	89.8 ± 10.9	93.2 ± 11.0	96.9 ± 11.0	< 0.001	a,b,c,d,e,f
FPG (mg/dl)	91.4 ± 9.8	85.8 ± 7.8	90.2 ± 8.3	92.9 ± 9.1	96.8 ± 10.3	< 0.001	a,b,c,d,e,f
Total cholesterol (mg/dL)	190.3 ± 33.3	175.7 ± 29.6	185.6 ± 29.7	195.5 ± 32.1	204.4 ± 34.4	< 0.001	a,b,c,d,e,f
Triglyceride (mg/dL)	124.2 ± 84.9	58.9 ± 10.7	87.3 ± 11.6	123.5 ± 17.9	227.9 ± 109.2	< 0.001	a,b,c,d,e,f
HDL-cholesterol (mg/dL)	53.2 ± 12.6	61.3 ± 12.4	55.8 ± 11.9	50.7 ± 10.7	45.1 ± 9.2	< 0.001	a,b,c,d,e,f
hsCRP (mg/L)	1.0 ± 1.3	0.7 ± 1.2	0.9 ± 1.3	1.2 ± 1.4	1.3 ± 1.4	< 0.001	a,b,c,d,e,f
Current smoker (%)	24.7	11.2	19.3	27.1	41.1	< 0.001	-
Alcohol drinking (%)	43.3	35.2	39.2	44.6	54.3	< 0.001	-
Regular exercise (%)	30.9	33.4	32.7	31.2	26.3	< 0.001	-
Hypertension (%)	20.3	9.1	15.5	23.4	33.5	< 0.001	-
Chronic kidney disease (%)	1.9	1.5	1.7	2.2	2.3	0.011	-

*BP* blood pressure, *FPG* fasting plasma glucose, *HDL* high-density lipoprotein, *hsCRP* high-sensitivity C-reactive protein

*P values were calculated using one-way ANOVA test or Pearson’s chi-square test

^†^ Post hoc analysis with Bonferroni method: a, Q1 versus Q2; b, Q1 versus Q3; c, Q1 versus Q4; d, Q2 versus Q3; e, Q2 versus Q4; and f, Q3 versus Q4

**Fig. 2:**
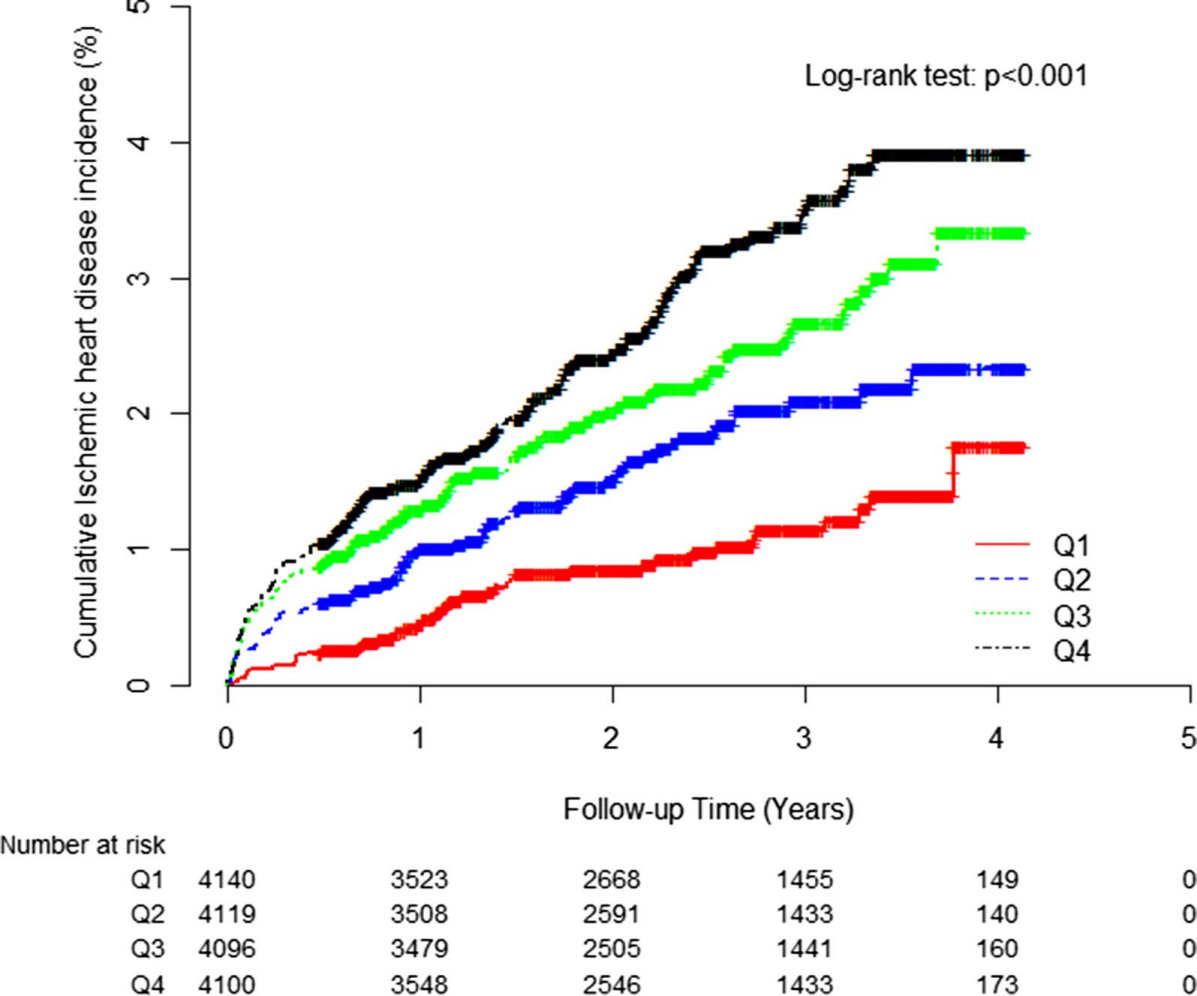
Kaplan–Meier plots indicating the cumulative probability of being diagnosed with ischemic heart disease after the baseline survey

Mean BMI, mean arterial pressure, total cholesterol, and hsCRP values were highest and mean HDL-cholesterol levels were lowest in the highest TyG index quartile group. The greatest proportions of current smokers and alcohol drinkers were members of the fourth TyG index quartile, whereas the proportion of individuals who participated in regular exercise was highest in the first TyG index quartile. The higher TyG index groups had a significantly elevated cumulative incidence of IHD over a 50-month period that followed the baseline survey (log-rank test, *P* < 0.001) (Fig. 2).

Using a pairwise comparison of ROC analyses of incident IHD, the AUC of TyG index data was significantly higher than that of fasting plasma glucose (*P* = 0.016) and was marginally significant when compared with the AUC produced using serum triglyceride level data (*P* = 0.058). The sensitivity, specificity, and AUC of the TyG index for classifying IHD were 80.8%, 37.2%, and 0.613, respectively (Table 2).

**Table 2:** TyG index versus fasting glucose and serum triglyceride levels for predicting ischemic heart disease

	Pairwise comparison of AUC			Ability to classify IHD			
	Difference	95% CI	*P* value	Sensitivity (%)	Specificity (%)	AUC	*P* value
TyG index vs. fasting plasma glucose	0.04	0.01 to 0.08	0.016				
TyG index vs. serum triglyceride levels	0.01	0.00 to 0.01	0.058				
Fasting plasma glucose vs. serum triglyceride levels	0.04	0.00 to 0.07	0.072				
TyG index				80.8	37.2	0.613	< 0.001
Fasting plasma glucose				50.0	70.7	0.571	< 0.001
Serum triglyceride levels				67.1	50.6	0.607	< 0.001

*TyG index* triglyceride-glucose index, *AUC* area under the receiver operating characteristic curve, *IHD* ischemic heart disease

**Fig. 3:**
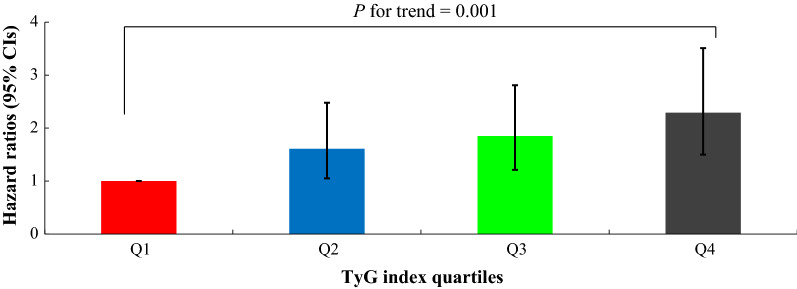
Hazard ratios (95% CIs) for incident IHD according to TyG index quartiles after adjusting for age, sex, body mass index, smoking status, alcohol intake, and physical activity

Table 3; Fig. 3 show results of the multivariate Cox proportional hazards regression analysis for the prediction of IHD according to TyG index quartile. A total of 322 individuals (2.0%, 322/16,455) developed IHD during the follow-up period. The incidence rate (per 1,000 person years) of IHD increased proportionally as TyG index quartile increased. Compared with the first TyG index quartile, the HRs of incident IHD for the second, third, and fourth quartiles increased in a dose-responsive manner. The HRs of incident IHD were 1.61 (95% CI 1.05–2.48), 1.85 (95% CI 1.21–2.83), and 2.28 (95% CI 1.48–3.51) for the second, third, and fourth TyG index quartiles, respectively, after adjusting for age, sex, BMI, smoking status, alcohol intake, physical activity, mean arterial blood pressure, hsCRP, CKD, and hypertension medication (Model 4).

**Table 3:** Hazard ratios and 95% confidence intervals for new-onset ischemic heart diseases according to TyG index quartiles

	TyG index quartiles				
	Q1 n = 4140	Q2 n = 4119	Q3 n = 4096	Q4 n = 4100	*P* for trend
New cases of ischemic heart disease, n	41	70	93	118	
Mean follow-up, years	2.4 ± 1.1	2.4 ± 1.1	2.4 ± 1.1	2.4 ± 1.1	
Pearson-years of follow-up	9878	9745	9651	9756	
Incidence rate/1000 person -years	4.2	7.2	9.6	12.1	
Model 1	1.00 (reference)	1.41 (0.96–2.08)	1.67 (1.15–2.42)	2.13 (1.48–3.06)	< 0.001
Model 2	1.00 (reference)	1.61 (1.05–2.48)	1.85 (1.21–2.81)	2.29 (1.50–3.51)	0.001
Model 3	1.00 (reference)	1.63 (1.06–2.49)	1.88 (1.23–2.87)	2.35 (1.53–3.61)	0.001
Model 4	1.00 (reference)	1.61 (1.05–2.48)	1.85 (1.21–2.83)	2.28 (1.48–3.51)	0.002

*TyG index* triglyceride-glucose index

^*^ Multivariate cox proportional-hazards regression model analysis

Model 1: adjusted for age and sex.

Model 2: adjusted for age, sex, body mass index, smoking status, alcohol intake, and physical activity.

Model 3: adjusted for age, sex, body mass index, smoking status, alcohol intake, physical activity, high sensitivity C-reactive protein, and mean arterial blood pressure, C-reactive protein level, and chronic kidney disease.

Model 4: adjusted for age, sex, body mass index, smoking status, alcohol intake, physical activity, high sensitivity C-reactive protein, mean arterial blood pressure, C-reactive protein level, chronic kidney disease, and hypertension medication.

## Discussion

Among community dwelling Korean adults without diabetes, we found that elevated TyG index values were positively and independently associated with IHD incidence in this large-scale, prospective cohort study that included a 50-month follow-up. Our study showed that the association between TyG index and IHD persisted after further adjustment for lifestyle factors, inflammation, and mean arterial blood pressure.

### Related studies

Recent meta-analysis of 13 cohort studies reported that higher TyG index values may significantly precede type 2 diabetes [20]. Meanwhile, the TyG index has also been shown to be associated with a higher prevalence of symptomatic coronary artery disease, along with metabolic and behavioral risk factors, and could be used as a marker for atherosclerosis [21]. Other studies have demonstrated significant associations between elevated TyG index values and a higher risk of arterial stiffness and nephric microvascular damage [22], between the TyG index and HOMA-IR [23] and subclinical myocardial injury [24], and between the TyG index and an increased risk of CVD incidence, especially among younger individuals [25].

In a prospective study that considered patients with stable coronary artery disease, Jin et al. showed that the TyG index may be a useful predictive marker of cardiovascular events [11]. Ma et al., in a longitudinal study of 766 patients who underwent percutaneous coronary intervention, reported that subgroups within the top tertile of the TyG index had a 2.17-fold higher risk of adverse cardiovascular outcomes over a median follow-up period of 30 months, compared with the referent first tertile [10]. Another cohort study by Sanchez-Inigo et al. examined the relationship between the TyG index and incident IHD in 5,014 Caucasian men and women with a mean age of 55.51 ± 13.68 years and 53.72 ± 12.84 years, respectively. Their work revealed a positive association between the TyG index and CVD over a median period of 10 years. However, the study performed by Sanchez-Inigo et al. included an older age group, patients with diabetes, and current users of anti-aggregation medications [26]. They were unable to identify a relationship between the TyG index and incident CVD in participants with type 2 diabetes at baseline, which may have been due to effects of medication or the adoption of heathier habits by participants [26].

Meanwhile, some studies have reported that the TyG index is associated with subclinical coronary atherosclerosis in both diabetic patients and the general population [13, 27]. From an epidemiological standpoint, nondiabetic individuals with myocardial infarction have been reported to have a poorer prognosis than diabetic patients without myocardial infarction [15, 16, 28, 29]. To predict future CVD, a health risk assessment over a 5-year period has become as important as that over a 10-year period over the past few decades [30, 31]. For the first time, our study revealed an association between the TyG index and incident IHD among nondiabetic adults in the preclinical stage using an assessment period that did not exceed 5 years in an East Asian population, despite the fact that the incidence of IHD was relatively low.

### Possible mechanisms

Some possible explanations for the observed association deserve consideration. The TyG index is considered one of the best indices for identifying individuals with early insulin resistance [3]. In a prospective study of non-obese Chinese adults, Zhang et al. suggested that the TyG index may be valuable for predicting type 2 diabetes [32]. Further, TyG index was determined to be superior to well-known predictive biomarkers of type 2 diabetes, such as HOMA-IR, in a study that assessed 5,354 Korean subjects without diabetes with a mean age of 61.6 years and a mean BMI of 24.2 kg/m^2^ [5]. Additionally, the TyG index has been suggested to be a useful surrogate marker of overall metabolic health status according to KNHANES, a nationwide survey representing the entire Korean population [33]. Finally, chronic inflammation could contribute to the association between the TyG index and IHD. In the present study, serum hsCRP levels gradually increased with TyG index quartile, which supports the idea that TyG index is closely linked to underlying low-grade inflammation.

### Study strengths and limitations

Some strengths and limitations require careful consideration and may affect the interpretation of the results of the present study. A major strength of the work was that we conducted a prospective cohort study using a large number of Korean individuals linked to HIRA data, which are derived from the universal coverage system in Korea. As a result, there was a very low chance that data were missing [34]. This study had some limitations that should also be acknowledged. First, because the study cohort was composed of volunteers that visited a clinic for health promotion screenings conducted at a single hospital, patients appeared to be slightly healthier than most community-based cohorts previously assessed, and some possible confounding variables may not have been measured at baseline. Second, some diabetic individuals may have been included in the study population because glycated haemoglobin A1c and 2-h oral glucose tolerance tests were not performed at the beginning of the study.

## Conclusions

In conclusion, an elevated TyG index precedes and significantly predicts future IHD among community dwelling nondiabetic Koreans. Moreover, the TyG index was found to be a more powerful predictive indicator of IHD than fasting glucose or triglyceride levels alone. Accordingly, a high TyG index may be a useful additional measure with which to assess cardiovascular risk for nondiabetic adults in the preclinical stage. Large-scale prospective studies are necessary to elucidate the mechanism for underlying the association between the TyG index and IHD.

## Data Availability

The datasets used and/or analysed in the current study are available from the corresponding author on reasonable request.

## Abbreviations

IHD: Ischemic heart disease
TyG: Triglyceride glucose
HERAS: Health risk assessment study
HIRA: Korea Health Insurance Review and Assessment
HRs: Hazard ratios
CIs: Confidence intervals
HOMA-IR: Homeostasis model assessment of insulin resistance
BMI: Body mass index
KNHANES: Korea National Health and Nutrition Examination Survey
CVD: Cardiovascular diseases
hsCRP: High sensitivity C-reactive protein
CKD: Chronic kidney disease
ANOVA: Analysis of variance
ROC: Receiver-operating characteristic
AUC: Area under the receiver-operating characteristic curve
